# Deformation Mechanisms Dominated by Decomposition of an Interfacial Misfit Dislocation Network in Ni/Ni_3_Al Multilayer Structures

**DOI:** 10.3390/ma17164006

**Published:** 2024-08-12

**Authors:** Zhiwei Zhang, Xingyi Zhang, Rong Yang, Jun Wang, Chunsheng Lu

**Affiliations:** 1 Department of Mechanics and Engineering Sciences, College of Civil Engineering and Mechanics, Lanzhou University, Lanzhou 730000, China; zhangxingyi@lzu.edu.cn; 2 Key Laboratory of Mechanics on Environment and Disaster in Western China, The Ministry of Education of China, Lanzhou University, Lanzhou 730000, China; 3 State Key Laboratory of Nonlinear Mechanics (LNM), Institute of Mechanics, Chinese Academy of Sciences, Beijing 100190, China; yangr@lnm.imech.ac.cn; 4 School of Civil and Mechanical Engineering, Curtin University, Perth, WA 6845, Australia; c.lu@curtin.edu.au

**Keywords:** Ni/Ni_3_Al multilayer structures, interfacial misfit dislocation network, dislocation evolution, crystalline orientation effect, molecular dynamics

## Abstract

Ni/Ni_3_Al heterogeneous multilayer structures are widely used in aerospace manufacturing because of their unique coherent interfaces and excellent mechanical properties. Revealing the deformation mechanisms of interfacial structures is of great significance for microstructural design and their engineering applications. Thus, this work aims to establish the connection between the evolution of an interfacial misfit dislocation (IMD) network and tensile deformation mechanisms of Ni/Ni_3_Al multilayer structures. It is shown that the decomposition of IMD networks dominates the deformation of Ni/Ni_3_Al multilayer structures, which exhibits distinct effects on crystallographic orientation and layer thickness. Specifically, the Ni/Ni_3_Al (100) multilayer structure achieves its maximum yield strength of 5.28 GPa at the layer thickness of 3.19 nm. As a comparison, the (110) case has a maximum yield strength of 4.35 GPa as the layer thickness is 3.01 nm. However, the yield strength of the (111) one seems irrelevant to layer thickness, which fluctuates between 10.89 and 11.81 GPa. These findings can provide new insights into a deep understanding of the evolution and deformation of the IMD network of Ni/Ni_3_Al multilayer structures.

## 1. Introduction

Ni-based superalloys have been widely utilized to manufacture aircraft engine blades in aerospace industries due to their desirable mechanical properties and resistance to creep, corrosion, and oxidation [1,2]. These excellent thermomechanical properties are mainly attributed to the superlattice structure of Ni_3_Al precipitates and the interfacial misfit dislocation (IMD) network between Ni matrix and Ni_3_Al precipitates [3,4,5]. More recently, in addition to studies on the mechanical properties and deformation mechanisms of Ni_3_Al precipitates [6,7], there have been growing interests that are focused on the IMD network. This is mainly because, by absorbing and accommodating slip dislocations in the Ni matrix, the IMD network can impede dislocations from approaching or shearing into the Ni_3_Al phase. Especially when applied in multilayer structures, it may play a more important role in dominating deformation [8,9].

Over the past few decades, numerous experiments and theoretical analyses have been carried out to ascertain the microstructural evolution and mechanical behaviors in Ni/Ni_3_Al heterogeneous multilayer structures [10,11,12,13,14]. For example, Zhang et al. [10] prepared Ni/Ni_3_Al nanostructured multilayers by magnetron sputtering and studied the microstructure and hardness of Ni/Ni_3_Al multilayers by transmission electron microscopy and nanoindentation. Sun et al. [11] investigated the phase stability of the Ni/Ni_3_Al multilayer structure under high-temperature annealing and irradiation. Yu et al. [12] performed molecular dynamics (MD) simulations to analyze the intergranular and transgranular crack propagation behavior at the Ni/Ni_3_Al interface. Shang et al. [13] explored the plastic deformation mechanism of the Ni/Ni3Al interface dislocation network with pre-voids under tensile loading and found out that the main plastic deformation is due to the propagation of slip bands emitted from stair-rod dislocation and stacking faults generated from Shockley partials. Liu et al. [14] also applied MD simulations to examine the microstructure and properties of thin Ni/Ni_3_Al under uniaxial tension of twist grain boundaries. Hocker et al. [15] studied brittle/ductile interfaces of Ni/NiAl under mechanical loading and their results showed that interfaces have an influence on strain-induced material failure through nucleation of defects. Although relevant experiments have captured the IMD in Ni/Ni_3_Al heterogeneous multilayer structures, its evolution process during deformation is rather elusive. However, it is fortunate that such a missing process can be addressed via MD simulations. Here, to the best of our knowledge, there are still no reports on the evolution of the IMD network within Ni/Ni_3_Al heterogeneous multilayer structures with various crystallographic orientations under deformation.

In this paper, to establish the connection between the evolution of the IMD network and tensile deformation mechanisms of Ni/Ni_3_Al multilayer structures, the MD simulation is adopted to comprehensively investigate the evolution of the IMD network within Ni/Ni_3_Al heterogeneous multilayer structures under tensile deformation along various crystallographic orientations. The length of dislocations and microstructural evolution are extracted to clarify the deformation mechanism of Ni/Ni_3_Al heterogeneous multilayer structures. In addition, the effect of layer thickness is elaborated and studied on the yield strength of Ni/Ni_3_Al multilayer structures.

## 2. Numerical Models and Methodology

### 2.1. Ni/Ni_3_Al Heterogeneous Interface Models

**Figure 1:**
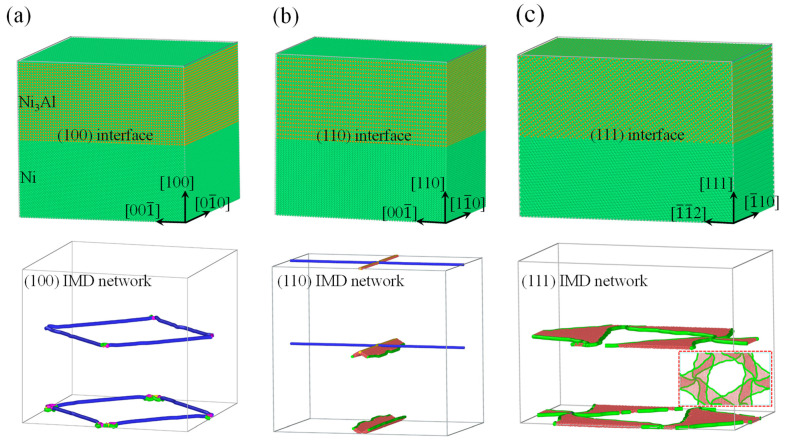
The heterogeneous interfacial configurations and IMD networks of Ni/Ni_3_Al multilayer structures with various crystallographic orientations. (**a**) (100) Ni/Ni_3_Al interface and (100) IMD network, (**b**) (110) Ni/Ni_3_Al interface and (110) IMD network, and (**c**) (111) Ni/Ni_3_Al interface and (111) IMD network, where atoms are colored by dislocation analysis with red representing stacking faults and green, blue, and yellow lines indicating 1/6<112> Shockley, 1/2<110> perfect and 1/3<100> Hirth dislocations, respectively. Inset in (**c**) shows a bottom view of the IMD network structure. FCC structures are removed for clarity.

As illustrated in Figure 1, the heterogeneous interfacial configurations of Ni/Ni_3_Al multilayer structures were constructed with various crystallographic orientations. As is well known, lattice misfit corresponds to the deformation of an invariant lattice. Let us take the Ni/Ni_3_Al (100) heterogeneous interfacial configuration as an example. Considering the size coincidence of two heterogeneous lattices on the (100) misfit interphase interface, there are at least 66 Ni_3_Al lattices and 67 Ni lattices to relax stress induced by the difference in lattice parameters (3.52 Å for Ni and 3.573 Å for Ni_3_Al) [16,17]. The smallest size of the Ni/Ni_3_Al (100) heterogeneous interface is obtained as 3.52 × 67 ≈ 3.573 × 66 ≈ 23.6 nm. Here, the volume of the Ni/Ni_3_Al (100) multilayer structure is 23.6 × 23.6 × 21.3 nm^3^, consisting of more than 0.9 million atoms (see Figure 1a). It is shown that semi-coherent square Ni/Ni_3_Al IMD networks form on interphase interfaces to reduce the distortion energy of the system. It is also seen that a Ni/Ni_3_Al (100) square IMD network mainly contains four 1/2<110> perfect dislocations.

Similarly, the Ni/Ni_3_Al (110) and Ni/Ni_3_Al (111) heterogeneous interfacial configurations were constructed, with volumes of 16.7 × 23.6 × 25.1 nm^3^ and 16.7 × 28.9 × 30.7 nm^3^, respectively (see Figure 1b,c). After energy minimization, to accommodate the misfit strain due to the lattice difference between the two phases, the regular quadrilateral and equilateral triangular IMD networks form on the (110) and (111) interfaces of Ni/Ni_3_Al, as shown in Figure 1b,c, respectively. Among them, the Ni/Ni_3_Al (110) semi-coherent IMD network consists of one 1/2[0$\overset{-}{1}$1] perfect dislocation and one 1/3[100] Hirth dislocation at the interface. The latter connects to one 1/6[2$\overset{-}{1}$1] and one 1/6[21$\overset{-}{1}$] Shockley dislocation with two segments of stacking faults in the Ni matrix. The Ni/Ni_3_Al (111) IMD network consists of three equilateral triangle regions of stacking faults with three 1/6<112> Shockley dislocations as their edges. In addition, various layer thicknesses were constructed to investigate their effects on the mechanical properties of Ni/Ni_3_Al multilayer structures.

### 2.2. Molecular Dynamics Simulation

Atomistic simulations were performed by using the Largescale Atomic/Molecular Massively Parallel Simulator [18]. An embedded-atom potential function for the Ni-Al system developed by Mishin [19] was taken to describe the atomic interaction in Ni_3_Al and Ni multilayer structures. In this function, the total energy, *E*, of a system is represented by

(1) $$E=\sum_{\begin{array}{l} i,j\\ i\neq j \end{array}}V_{EAM}(r_{ij})+\sum_{i}F(\bar{\rho_{i}})$$

where *V*_EAM_(*r_ij_*), a pair potential, is a function of the distance *r_ij_* between atoms *i* and *j*. Moreover, *F* is the embedding energy of atom *i* and $\bar{\rho_{i}}$ is the electron density, which is defined as

(2) $$\bar{\rho_{i}}=\sum_{i\neq j}g_{j}(r_{ij})$$

where $g_{j}(r_{ij})$ is the electron density of atom *j*.

Such a potential was built up by fitting to data of both experiments and first principles. It can be applied to depict an accurate lattice, the mechanical properties, and the energetics of point defects and planar faults especially. The latter is essential to study planar fault dominated deformation mechanisms of Ni_3_Al [20,21]. In addition, periodic boundary conditions were introduced in three directions and initial configurations were energetically minimized by relaxing all samples for 100 ps at 300 K with the Nosé–Hoover thermostat [17]. Simulations were performed by integrating Newton’s equations of motion for all atoms with a time step of 1 fs. To obtain the mechanical properties, a constant strain rate of 5 × 10^8^ s^−1^ was applied. The comparison between MD simulation results and that of experiments can be made with the help of a theoretical model involving strain rate and interface dimension given that experimental data are available [22]. Here, we have to concentrate on simulation results due to lack of experimental ones. Generally, crystallographic orientations are too many to list all their effects on the mechanical properties of a crystal. Here, three common orientations, [100], [110] and [111], were chosen to investigate the orientation dependent mechanical properties of Ni/Ni_3_Al multilayer structures. The first one involves a uniaxial tensile load along the [100] and [010] directions for the Ni/Ni_3_Al (100) heterogeneous interfacial configuration. The second one has two cases along [110] and [1$\overset{-}{1}$0] directions for the Ni/Ni_3_Al (110) heterogeneous interfacial configuration. However, the third one only possesses the [111] tensile direction for the Ni/Ni_3_Al (111) heterogeneous interfacial configuration. Stress in a stress–strain relationship was calculated by the Virial scheme [23,24,25], which depicts the average stress *σ* over a volume *Ω* around an atom *i* with mass *m_i_* and velocity ***v****_i_* at position ***r****_i_* subjected to force ***f****_ij_* from atom *j* as

(3) $$\sigma =\frac{1}{\Omega }(-m_{i}v_{i}\times v_{i}+\frac{1}{2}\sum_{j(\neq i)}r_{ij}\times f_{ij}).$$

During uniaxial loading, deformation and defects of Ni/Ni_3_Al heterogeneous interfacial configurations were recognized by common neighbor and dislocation analysis and then visualized with the software OVITO [26].

## 3. Results

### 3.1. Ni/Ni_3_Al (100) Interface

**Figure 2:**
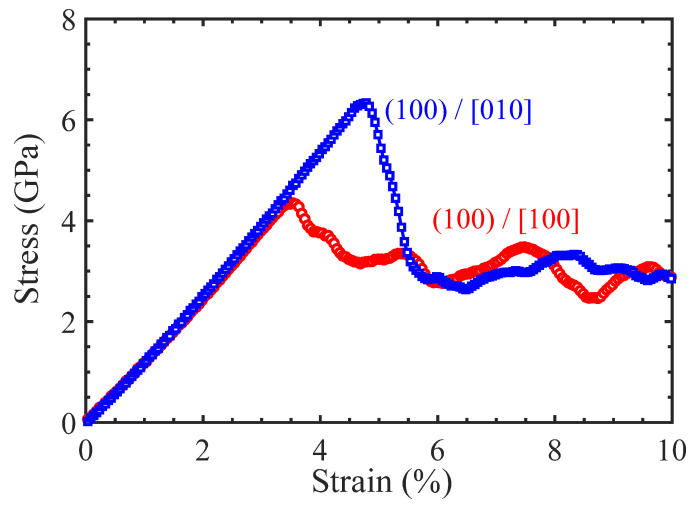
The tensile stress–strain curves of the Ni/Ni_3_Al (100) heterogeneous multilayer structure with tensile loading along the [100] and [010] orientations.

As shown in Figure 2, the tensile stresses along the [100] and [010] crystalline directions in the Ni/Ni_3_Al (100) heterogeneous interfacial configuration linearly increase with strain until the yield strengths. The former produces the yield strength of 4.35 GPa at strain 3.5%, which are smaller than that verified in the latter (the yield strength of 6.34 GPa at strain 4.8%). Subsequently, as strain further increases, the stress–strain curves in both cases firstly drop and then enter a plastic flow stage.

**Figure 3:**
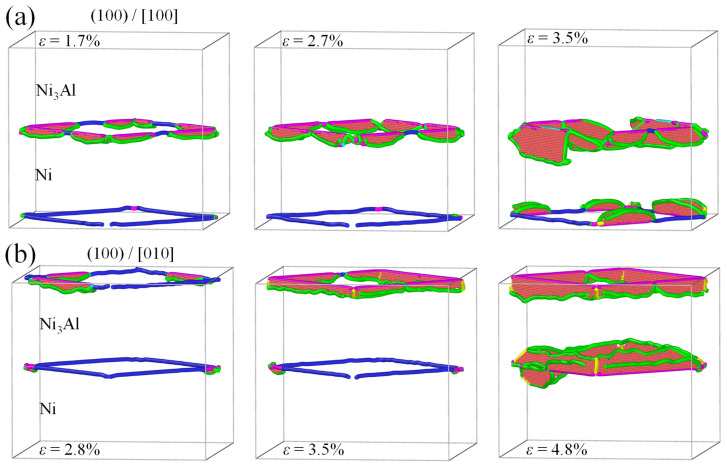
The microstructural evolution and decomposition of the Ni/Ni_3_Al (100) IMD network with loading along (**a**) [100] and (**b**) [010] orientations at various strains, where atoms are colored by dislocation analysis with red representing stacking faults. Green, blue, purple, and yellow lines indicating 1/6<112> Shockley, 1/2<110> perfect, 1/6<110> stair-rod and 1/3<100> Hirth dislocations, respectively. FCC structures are removed for clarity.

**Figure 4:**
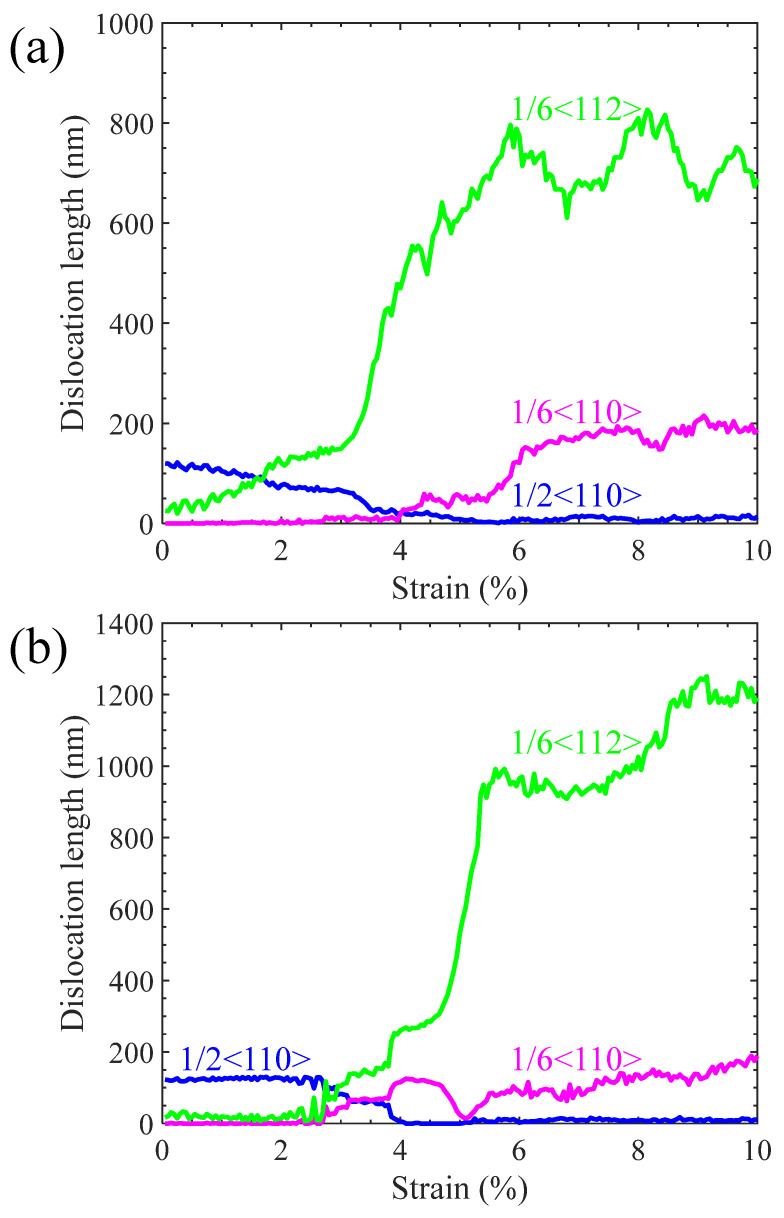
Lengths of 1/6<112>, 1/6<110>, and 1/2<110> dislocations vary with strain as the Ni/Ni_3_Al (100) heterogeneous multilayer structure is stretched along (**a**) [100] and (**b**) [010] orientations.

Real-time detection on activities of dislocations and stacking faults indicates that the yield strength is closely related to their evolution, as illustrated in Figure 3. In the case of loading along the [100] crystalline orientation, one 1/2<110> perfect dislocation segment from an IMD network on one Ni/Ni_3_Al (100) heterogeneous interface is decomposed to two 1/6<112> Shockley dislocations and one 1/6<110> stair-rod dislocation at strain of 1.7%. Each 1/6<110> stair-rod dislocation connects with a 1/6<112> Shockley dislocation by a stacking fault between them in the Ni matrix (see Figure 3a). With the increase in strain, more 1/2<110> perfect dislocations decompose to form 1/6<112> Shockley dislocations, 1/6<110> stair-rod dislocations, and stacking faults. The decomposing reaction can be written as 1/2<110> → 1/6<112> + 1/6<112> + 1/6<110>. It is worth noting, however, that when the Ni/Ni_3_Al (100) heterogeneous interfacial configuration reaches the yield stress, stacking faults and dislocations are concentrated in the Ni matrix. The dislocation evolution can be quantified by variation in dislocation lengths with strain (see Figure 4a). As strain increases, the lengths of 1/2<110> perfect dislocations gradually decrease. On the contrary, growth is observed on lengths of 1/6<112> Shockley and 1/6<110> stair-rod dislocations. At the yield point, the lengths of 1/2<110> perfect dislocations are close to zero, demonstrating that all the 1/2<110> perfect dislocations in the IMD network at the Ni/Ni_3_Al (100) heterogeneous interfaces are decomposed into 1/6<112> Shockley and 1/6<110> stair-rod dislocations. After the yield point, 1/6<112> Shockley and 1/6<110> stair-rod dislocations multiply sharply and account for the main dislocation components with increasing the tensile strain.

In the case of loading along the [010] crystalline orientation, the same dislocation decomposing reaction (Figure 3b) and trend of length of dislocations are seen with variation in strain (Figure 4b). However, at the yield point, dislocations and stacking faults are mainly accumulated in the Ni_3_Al layer. Only a small stacking fault tetrahedron is found in the Ni matrix.

### 3.2. Ni/Ni_3_Al (110) Interface

**Figure 5:**
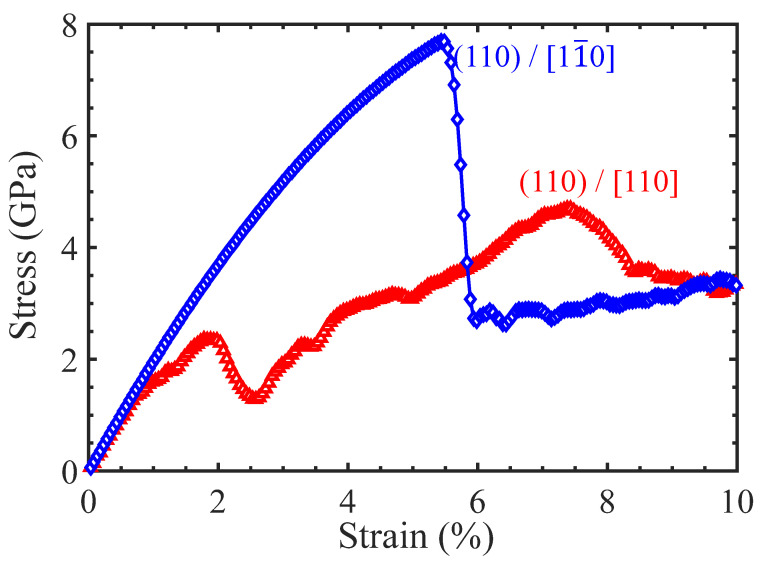
The tensile stress–strain curves of the Ni/Ni_3_Al (110) heterogeneous multilayer structure with tensile loading along [110] and [1$\overset{-}{1}$0] orientations.

**Figure 6:**
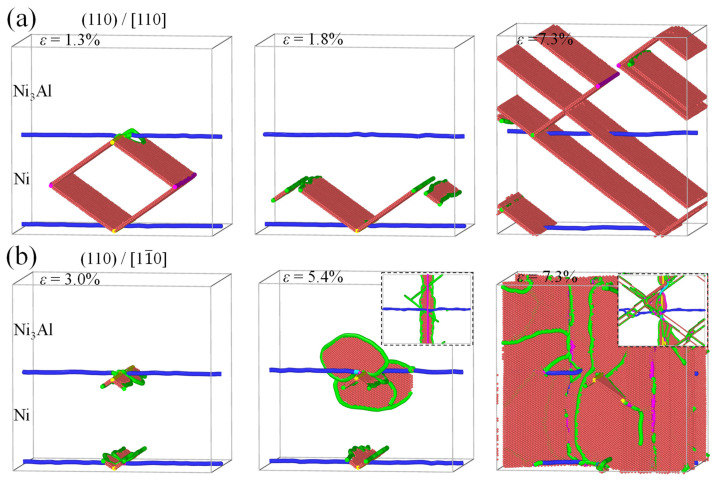
The evolution of the Ni/Ni_3_Al (110) IMD network with loading along (**a**) [110] and (**b**) [1$\overset{-}{1}$0] orientations at various strains, where atoms are colored by dislocation analysis with red representing stacking faults and green, blue, purple, and yellow lines indicating 1/6<112> Shockley, 1/2<110> perfect, 1/6<110> stair-rod, and 1/3<100> Hirth dislocations, respectively. FCC structures are removed for clarity. Insets in (b) show bottom views of dislocation structures.

**Figure 7:**
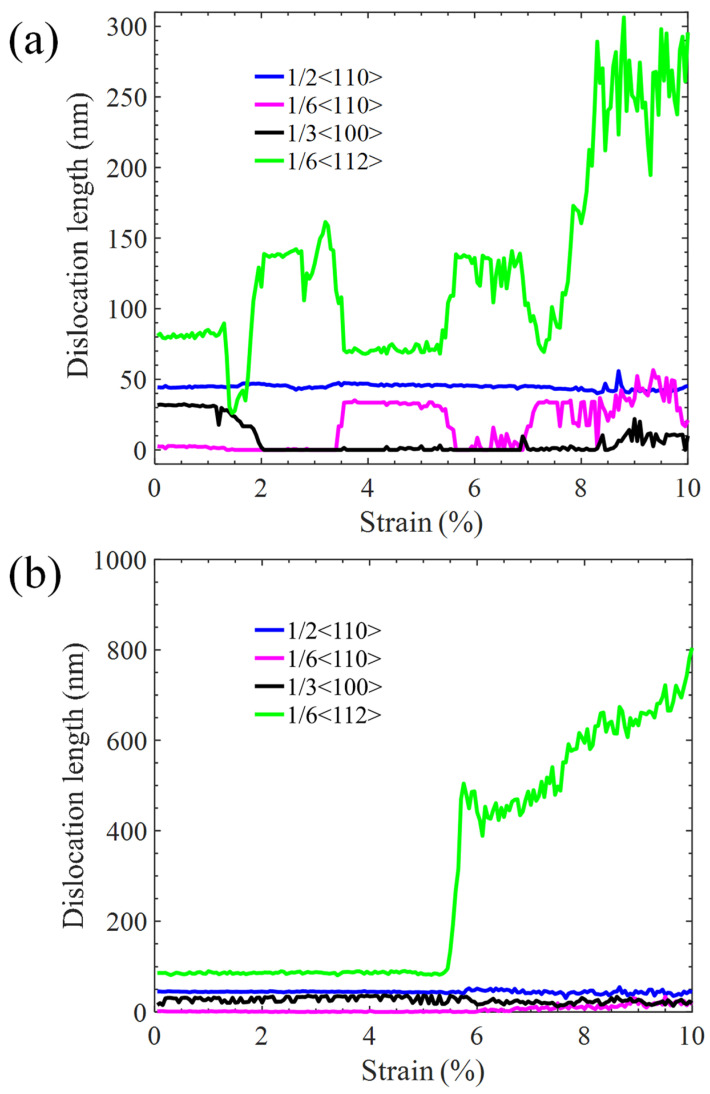
Lengths of 1/6<112>, 1/6<110>, 1/3<110>, and 1/2<110> dislocations vary with strain as the Ni/Ni_3_Al (110) heterogeneous multilayer structure is stretched along (**a**) [110] and (**b**) [1$\overset{-}{1}$0] orientations.

Figure 5 shows the tensile stress–strain curves of the Ni/Ni_3_Al (110) heterogeneous interfacial configuration with tensile loading along the [110] and [1$\overset{-}{1}$0] crystalline orientations, respectively. As the loading direction is along the [110] crystalline orientation, the stress–strain curve exhibits fluctuation before the maximum stress is achieved. Structural analysis shows that the four stacking fault segments (see Figure 1b) in the Ni/Ni_3_Al (110) IMD network expand into the Ni matrix. At the strain of 1.3%, their mutual contact causes dislocation reactions (1/6<112> + 1/6<112> → 1/6<110>) to form two 1/6<110> stair-rod dislocations. This brings a stacking fault rhombus cylinder (see Figure 6a). The dislocation reaction is reconfirmed from the opposite change in length between 1/6<112> Shockley and 1/6<110> stair-rod dislocations (see Figure 7a). As stress reaches its local peak of 2.43 GPa, the stacking fault rhombus cylinder decomposes. Each 1/6<110> stair-rod dislocation decomposes into two 1/6<112> Shockley dislocations, which expand toward the edge of the Ni matrix with stacking faults behind them. Subsequently, as strain increases to 7.3%, stress reaches its maximum value of 4.77 GPa. At this point, stacking faults cross the Ni/Ni_3_Al (110) interface and coexist in Ni and Ni_3_Al layers. During the entire deformation process, the 1/2<110> perfect dislocations remain motionless.

However, under loading along the [1$\overset{-}{1}$0] crystalline orientation, the tensile stress–strain curve linearly rises until the yield strength (7.65 GPa at the stain of 5.4%) is reached (see Figure 5). Below the yield point, there is no obvious dislocation reaction in the Ni/Ni_3_Al (110) IMD network, declaring its elastic deformation (Figure 6b). Beyond the yield point, stress drops sharply and then tends to a stable plastic flow state. Structural analysis reveals that 1/6<112> Shockley dislocations initiated from the Ni/Ni_3_Al (110) IMD network expand and multiply along the slip direction of [110] crystalline orientation. Finally, as strain ascends, stacking faults vertically penetrate the entire Ni/Ni_3_Al (110) heterogeneous interface configuration. The structural analysis is echoed by the evolution of dislocation lengths with varying strain (see Figure 7b).

### 3.3. Ni/Ni_3_Al (111) Interface

**Figure 8:**
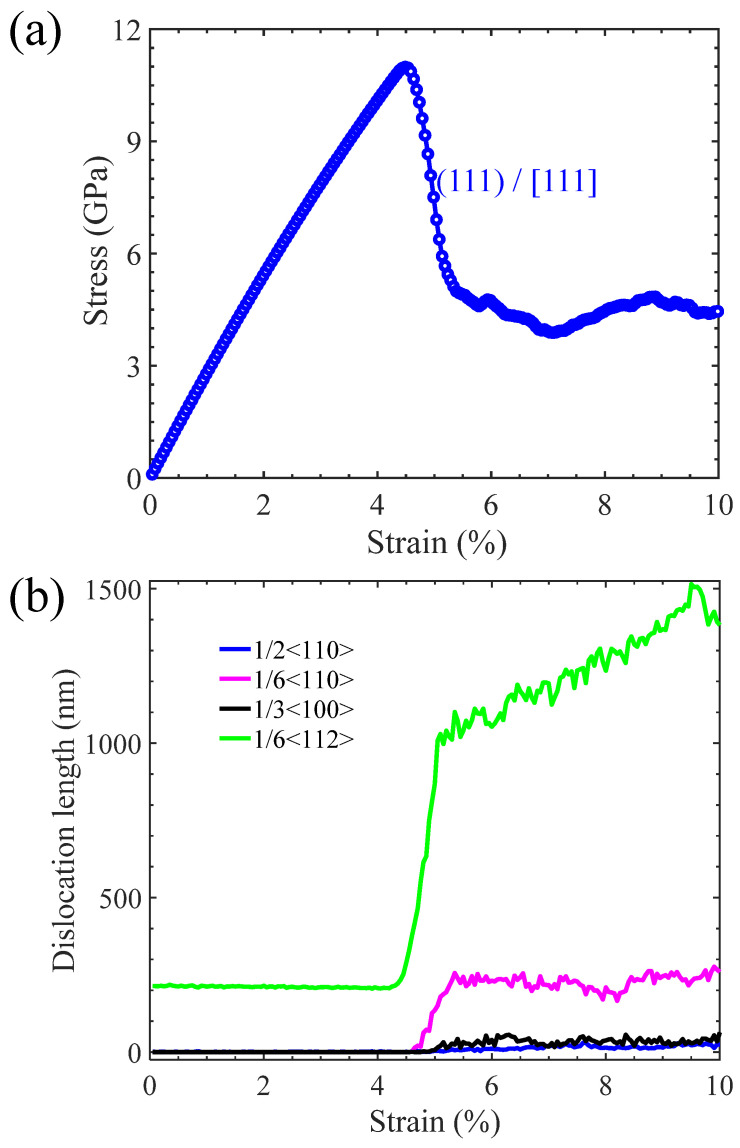
(**a**) The stress–strain curve of the Ni/Ni3Al (111) heterogeneous multilayer structure with tensile loading along the [111] orientation and (**b**) lengths of 1/6<112>, 1/6<110>, 1/3<110>, and 1/2<110> dislocations versus strain.

Figure 8a shows the tensile stress–strain relationship of the Ni/Ni_3_Al (111) heterogeneous interfacial configuration with loading along the [111] crystalline orientation. After stress reaches its peak value of 11.34 GPa at strain 4.5%, it drops and then tends to the plastic flow stage with a further increase in strain. Dislocation analysis in Figure 8b indicates that lengths of various dislocations remain unchanged before strain 4.5%, demonstrating the elastic deformation of the Ni/Ni_3_Al (111) heterogeneous interfacial configuration. However, after the yield point, 1/6<112> Shockley and 1/6<110> stair-rod dislocations increase sharply with the increase in strain. Then, the length of 1/6<110> stair-rod dislocations tends to be stable, while that of 1/6<112> Shockley dislocations is still in the process of proliferation.

**Figure 9:**
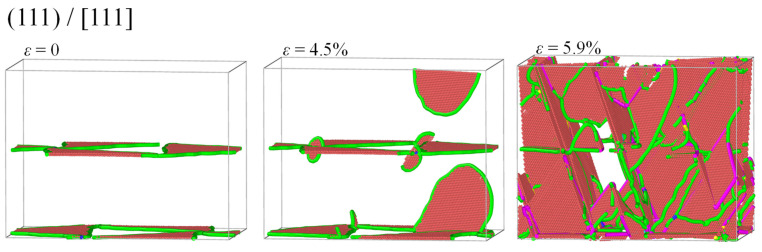
The Ni/Ni_3_Al (111) IMD network evolves with strain as tensile loading is along the [111] orientation, where atoms are colored with red representing stacking faults and green, blue, purple, and yellow lines indicating 1/6<112> Shockley, 1/2<110> perfect, 1/6<110> stair-rod, and 1/3<100> Hirth dislocations, respectively. FCC structures are removed for clarity.

From the perspective of microstructural evolution, it is also seen that, before the yield point (strain 4.5%), the Ni/Ni_3_Al (111) IMD network remains intact with its triangular pattern almost unchanged. However, new 1/6<112> Shockley dislocations originate at the triangle corner as soon as the yield point is reached. The newly generated 1/6<112> Shockley dislocations expand to the basic interior and drag a section of stacking faults behind them. Then, as newly generated 1/6<112> Shockley dislocations meet, 1/6<112> + 1/6<112> + 1/6<112> → 1/6<110> merging reaction occurs, forming a 1/6<110> stair-rod dislocation. After saturation of the merging reaction, the total amount of 1/6<110> stair-rod dislocations remains stable even with the additional multiplication of 1/6<112> Shockley dislocations (see Figure 9).

### 3.4. Effects of Layer Thickness for Ni/Ni_3_Al Multilayer Structure

**Figure 10:**
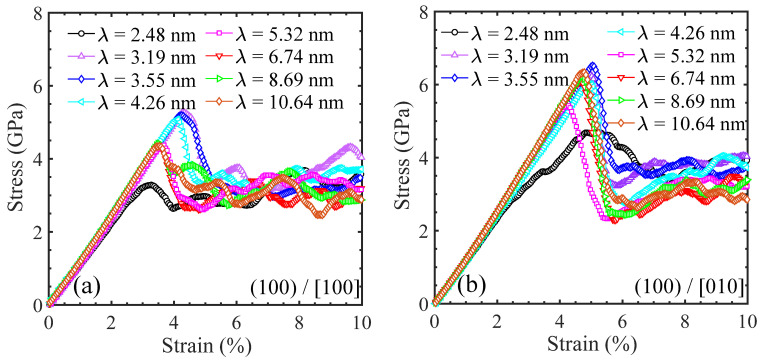
Tensile stress–strain curves of Ni/Ni_3_Al (100) heterogeneous multilayer structures with various layer thicknesses corresponding to loading along (**a**) [100] and (**b**) [010] orientations, respectively.

**Figure 11:**
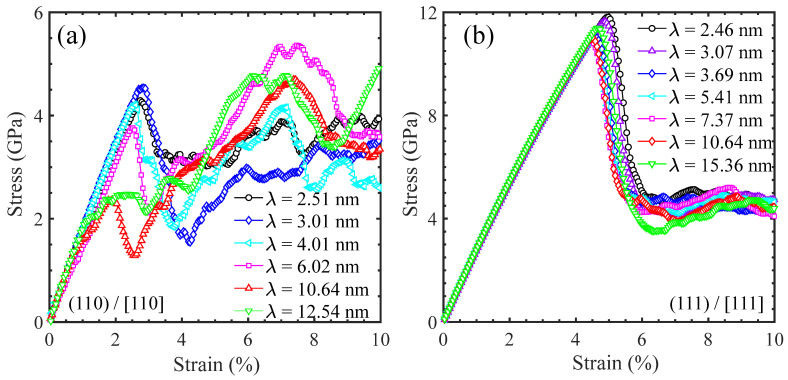
Tensile stress–strain curves of (**a**) Ni/Ni_3_Al (110) and (**b**) Ni/Ni_3_Al (111) heterogeneous multilayer structures vary with layer thickness as loading is along [110] and [111] orientations, respectively.

**Figure 12:**
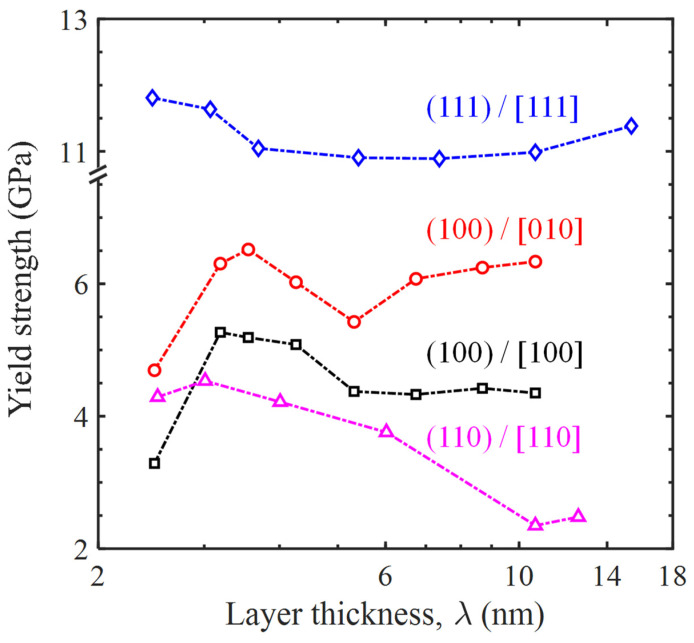
Dependence of yield strength of Ni/Ni_3_Al heterogeneous multilayer structures on their layer thicknesses.

A series of MD tensile simulations were performed to better understand the effect of layer thickness on the yield strength and deformation mechanisms of Ni/Ni_3_Al multilayer structures with the three common crystalline orientations. Specifically, the Ni/Ni_3_Al (100) heterogeneous interfacial configurations involve two loading directions and their stress–strain curves are shown in Figure 10. However, the Ni/Ni_3_Al (110) and Ni/Ni_3_Al (111) heterogeneous interfacial configurations only include the situation of stretching perpendicular to the (110) and (111) interface and their stress–strain curves are seen in Figure 11. It is shown that the yield strength of Ni/Ni_3_Al multilayer structures significantly depends on the layer thickness between Ni and Ni_3_Al layer matrix, as summarized in Figure 12. For the Ni/Ni_3_Al (100) heterogeneous interfacial configuration with loading along the [100] orientation, its yield strength rises from 3.29 to 5.28 GPa as the layer thickness increases from 2.48 to 3.19 nm and then gradually reduces and stabilizes around 4.35 GPa as the layer thickness further goes up. As loading is along the [010] orientation, the overall level of strength is higher although a similar trend is observed between strength and layer thickness.

The Ni/Ni_3_Al (110) heterogeneous interfacial configuration with loading along the [110] orientation also produces a first ascendant in strength from 4.29 to 4.35 GPa as the layer thickness increases from 2.51 to 3.01 nm. Similarly, strength then drops to 2.48 GPa with the growth of layer thickness to 12.54 nm. However, the Ni/Ni_3_Al (111) heterogeneous interfacial configuration with loading along the [111] orientation generates a roughly unchanged strength between 10.89 and 11.81 GPa with a layer thickness between 2.46 and 15.36 nm. The variation in the yield strength in the Ni/Ni_3_Al (111) heterogeneous interfacial configuration is relatively small compared with the other three cases.

**Figure 13:**
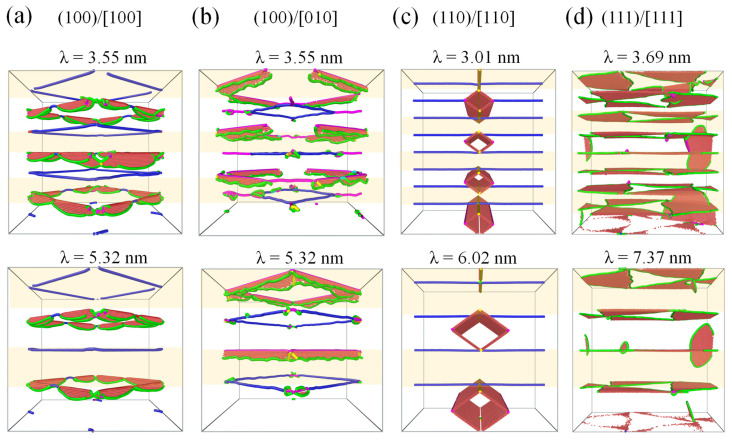
Microstructural evolution of Ni/Ni_3_Al heterogeneous multilayer structures with different layer thicknesses. The Ni/Ni_3_Al (100) multilayer structure is stretched along (**a**) [100] and (**b**) [010] orientations. (**c**) The Ni/Ni_3_Al (110) multilayer structure is tensibly loaded along the [110] orientation, while that for (**d**) the Ni/Ni_3_Al (111) multilayer structure is along the [111] orientation. Atoms are colored red representing stacking faults. Green, blue, purple, and yellow lines indicate 1/6<112> Shockley, 1/2<110> perfect, 1/6<110>, and 1/3<100> Hirth dislocations, respectively. FCC structures are removed for clarity.

Figure 13a,b shows the structural deformation of the Ni/Ni_3_Al (100) heterogeneous interfacial configuration with loading along the [100] and [010] orientation at the yield point with the layer thickness of 3.55 and 5.32 nm, respectively. In the case of 3.55 nm, the decomposition of IMD networks causes the spread of dislocations and stacking faults behind them in both Ni and Ni_3_Al layers. However, as the layer thickness is beyond 3.55 nm, only one IMD network from a layer decomposes at the yield point. This leads to the spread of dislocations and stacking faults within either Ni or Ni_3_Al layer. Figure 13c illustrates that the formation of stacking fault rhombus cylinders is within the Ni layers as loading is along the [110] orientation for the Ni/Ni_3_Al (110) heterogeneous interfacial configuration. However, propagation of 1/6<112> Shockley dislocations in the (111) IMD networks extends to both sides of the (111) interface as the Ni/Ni_3_Al (111) heterogeneous interfacial configuration is stretched along the [111] orientation (see Figure 13d).

## 4. Discussion

As mentioned above, structures of the IMD network at three main Ni/Ni_3_Al heterogeneous interfaces were obtained through MD simulation. For the Ni/Ni_3_Al (100), (110), and (111) interfaces, the IMD networks are square, regular quadrilateral, and equilateral triangular, respectively, which are consistent with the previous findings [27,28,29]. Tensile simulation further reveals that the yield strength of Ni/Ni_3_Al heterogeneous multilayer structures is significantly structural and orientation-dependent. It is shown that, for the same layer thickness of ~10 nm, the Ni/Ni_3_Al (110) multilayer structure produces the lowest yield strength, while the Ni/Ni_3_Al (111) one outputs the maximum value. The medium outcome is achieved by the Ni/Ni_3_Al (100) multilayer structure (Figure 12). This can be attributed to the order of difficulty in decomposing an IMD network at the Ni/Ni_3_Al interface. Specifically, decompositions for the (110), (100), and (111) cases follow the order of propagation of pre-existing 1/6<112> Shockley dislocations, decomposition of 1/2<110> perfect dislocations, and generation of new 1/6<112> Shockley dislocations. Driving a pre-existing dislocation is the easiest task while generating a new one is the hardest. This order determines the external effort to decompose an IMD network and thus gives the order of yield strength of a Ni/Ni_3_Al multilayer structure. This result also echoes that in single-crystal silicon [27] and Ni-based superalloy [28] crystals as the orientation effect shows.

The trend of yield strength varying with layer thickness can be explained by the Hall-Petch effect [29], which describes the inverse relation between strength and grain size. Here, the layer thickness plays the role of grain size. With the reduction in layer thickness, the space for dislocation activities is weakened and this induces the growth of yield strength. However, there is a turning point at a layer thickness of 3.55 nm for the Ni/Ni_3_Al (100) multilayer structure (Figure 12). Below the size, yield strength drops as the layer thickness further reduces. The turning point can be elucidated by the switch of dislocation activities. Li et al. [30] have shown that the conventional strengthening mechanism by dislocation pile-up and cutting through twin planes switch to the softening mechanism by twin-boundary migration as the grain size is below a certain value. Figure 13a,b shows that the dislocation activity switches from the decomposition of all IMD networks to 1/2 of them as the layer thickness is over 3.55 nm. Since the existence of an IMD network can hinder dislocation activities [31,32], their disappearance is an undoubted softening mechanism and thus leads to a drop in yield strength. The yield strength-layer thickness trend obtained here is qualitatively consistent with the previous experimental results [10]. However, no obvious turning point can be detected for the Ni/Ni_3_Al (110) and (111) multilayer structures due to the lack of the switch of dislocation activities.

## 5. Conclusions

In this paper, a series of MD simulations have been performed to investigate the evolution of the IMD networks and tensile deformation behaviors of Ni/Ni_3_Al heterogeneous multilayer structures with various crystallographic directions and layer thicknesses. It is shown that the decomposition of the IMD networks dominates the deformation of Ni/Ni_3_Al heterogeneous multilayer structures. The decomposition also depends on the loading direction and layer thickness. The main conclusions can be summarized as follows:

1. Whether the Ni/Ni_3_Al (100) interfacial configuration is tensibly loaded along the [100] or [010] orientation, it encounters the same 1/2<110> → 1/6<112> + 1/6<112> + 1/6<110> dislocation decomposition reaction. However, dislocations evolve in the Ni and Ni_3_Al layers for the two orientations, respectively;
2. As tensile loading is along the [110] orientation, decomposition of the Ni/Ni_3_Al (110) IMD network initiates from the propagation of 1/6<112> Shockley dislocations and stacking faults behind them. This causes the yielding of the Ni/Ni_3_Al (110) multilayer structure in advance in contrast to the case of loading, which is along the [1$\overset{-}{1}$0] orientation. The 1/2<110> perfect dislocations always remain stable, showing an insensitivity to loading orientations;
3. The decomposition of the Ni/Ni_3_Al (111) IMD network is derived from the generation of new 1/6<112> Shockley dislocations at its triangular corners. The newly formed 1/6<112> Shockley dislocations extend into both Ni and Ni_3_Al layers;
4. It is expected that these findings can provide new insights into a deep understanding of the evolution of IMD networks dominated deformation mechanism of Ni/Ni_3_Al heterogeneous multilayer structures and benefit their wide applications in the aerospace industry.

## Data Availability

The data presented in this study are available on request from the corresponding author.
